# Hydroxytyrosol isolation, comparison of synthetic routes and potential biological activities

**DOI:** 10.1002/fsn3.4349

**Published:** 2024-07-16

**Authors:** Enhui Wang, Yanfei Jiang, Chunyue Zhao

**Affiliations:** ^1^ Beijing Qingyan Boshi Health Management Co., Ltd Beijing China

**Keywords:** biocatalysis, biological activity, chemical synthesis, hydroxytyrosol, physical extraction

## Abstract

Hydroxytyrosol (HT) is a polyphenol found in the olive plant (*Olea europaea*) that has garnered attention from the food, feed, supplement, and pharmaceutical industries. HT has evolved from basic separation and extraction to chemical and biocatalytic synthesis. The yield of HT can reach 1.93 g/L/h through chemical synthesis and 7.7 g/L/h through biocatalysis; however, both methods are subject to inherent limitations. Furthermore, the potential health benefits associated with HT have been highlighted, including its ability to act as an antioxidant, reduce inflammation, combat cancer and obesity, and exert antibacterial and antiviral effects. Its neuroprotective effects, skin protection, and wound healing capabilities are also discussed. Given these remarkable biological properties, HT stands out as one of the most extensively investigated natural phenols. This review highlights future methods and pathways for the synthesis of HT, providing insights based on its bioactivity characteristics, health benefits, and potential future applications.

## Graphical abstract

Hydroxytyrosol (HT) is a polyphenol extracted from olive plant and its byproduct. This review highlights methods and pathways for the synthesis of hydroxytyrosol, providing insights based on its bioactivity characteristics, health benefits, and potential future applications.

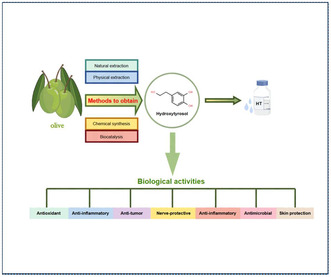

## 1 INTRODUCTION

**FIGURE 1:**
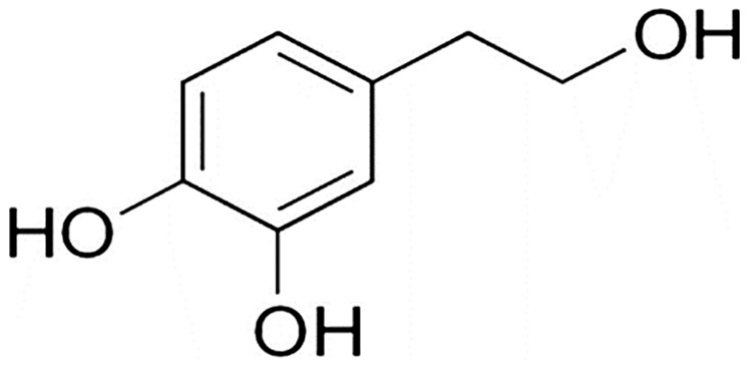
Hydroxytyrosol structure.

Hydroxytyrosol (HT) is a phenolic alcohol that is abundant in olive leaves and fruits (Stefanon & Colitti, 2016). The chemical formula of HT is C_8_H_10_O_3_, and the molecular weight is 154.164 g/mol. It is a compound that exhibits solubility in water and lipids. Its structure is shown in Figure 1.

The extraction of HT from olive or olive oil wastewater using organic solvents yields low concentrations, while the extraction and chromatography methods differ, resulting in significant environmental pollution (Achmon & Fishman, 2015). With the advancement of synthetic biology, both chemical and biological synthesis methods are now available for obtaining HT. Biotechnological synthesis offers a sustainable approach for HT production due to its low energy consumption, short growth period, and continuous advancements in genome editing technology. HT is produced at high levels by *Escherichia coli* and *Saccharomyces cerevisiae* using cost‐effective raw materials (Du et al., 2020; Liu et al., 2022). These acquisition methods are shown in Table 1.

**TABLE 1:** Comparison of different methods of obtaining hydroxytyrosol.

Acquisition	Source/method	Advantage	Disadvantage
From natural resources	Olives, grapes, and their byproducts, etc.	Sustainability	Limited availability; variability in concentration
Physical extraction	Solvent extraction; ultrasonic extraction; column chromatography separation	High purity; simple and easy; no expensive substrate	Low yield; high energy consumption; contain hazardous substances
Chemical synthesis	Baeyer‐Villager oxidation; extracted from clove oil, catechol, DOPAC, etc.	High purity; controllable; scalability	Expensive substrates; heavy metal pollution; harsh reaction condition
Biocatalytic production	Using enzymes or microorganisms to synthesize HT	High purity; more environmentally friendly; mild reaction conditions; more cost‐effective	Poor volumetric productivity; lower product titers

**FIGURE 2:**
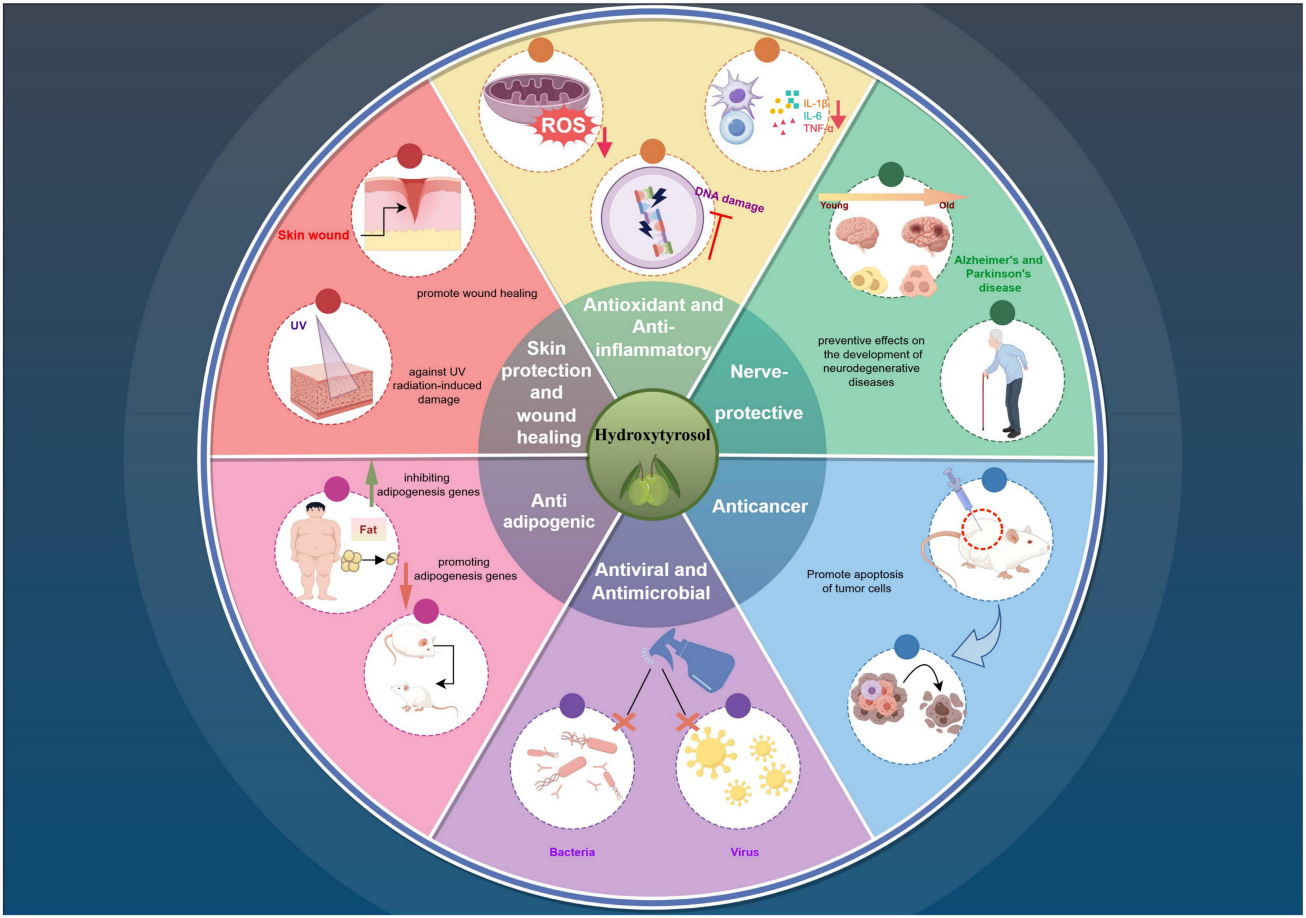
Biological activities of hydroxytyrosol.

Several studies have shown the beneficial effects of HT, which is a crucial polyphenolic compound found in the Mediterranean eating pattern, on health. These benefits encompass properties that combat inflammation, atherosclerosis, and thrombosis. Furthermore, HT has the ability to enhance endothelial function and combat steatosis (Stefanon & Colitti, 2016). Therefore, it is considered a neuroprotective, cardioprotective, and chemopreventive agent (Schaffer et al., 2007; Visioli, 2012; Visioli et al., 2004). Additionally, research suggests that HT may interact with proteins involved in cell cycle control and gene expression regulation exhibiting anticancer effects (Serreli & Deiana, 2018), as well as exhibiting certain antibacterial and antiviral effects (Bedoya et al., 2016). According to these results, HT has been listed as a nutritional supplement for preventing and treating various diseases (Table 2 and Figure 2).

**TABLE 2:** Biological function of hydroxytyrosol.

Pharmacological activity	Experimental model	Effect and pathway	Reference
Antioxidant	C57BL/6 mice; rats; pigment epithelial cells of the retina	The GSSG/GSH ratio in adipocytes was decreased; HT could enhance the electron transport chain; HT was able to increase the translocation of Nrf2 to the nucleus, which resulted in increased protein expression and activity of GCL, NQO1, HO‐1, GSH reductase, GSH peroxidase, and catalase; activity of the transcription factor forkhead transcription factor 3a	Forman et al. (2014), Fuccelli et al. (2018), Giordano et al. (2014), Granados‐Principal et al. (2014), Zhu et al. (2010), Zrelli, Matsuoka, Kitazaki, Araki, et al. (2011), Zrelli, Matsuoka, Kitazaki, Zarrouk, and Miyazaki (2011)
Anti‐inflammatory	Clinic trial; in vivo glioma model; SLE‐pristane induced model in BALB/c mice	It inhibits the inducible NOS/NO and cyclooxygenase/prostaglandin E2 pathways; inhibits the expression of inflammatory cytokines TNF‐α and IL‐1β; suppresses the activity and expression of MMP‐9 and COX‐2 enzymes in activated human monocytes; decreases the levels of pro‐inflammatory cytokines IL‐6	Borrie and Kim (2017), García‐García et al. (2021), Kapoor et al. (2014), Martínez et al. (2019), Ramírez‐Expósito and Martínez‐Martos (2018), Tavenier et al. (2020)
Anti‐cancer	MCF‐7 breast cancer cell; pancreatic, colon, prostate, thyroid, and leukemia cancer cells	HT to induce apoptosis; HT exerts its potential anticancer effect through the inhibition of Akt, NF‐κB, STAT3, and EGFR signaling pathways	Chimento et al. (2014), Fabiani et al. (2002), Goldsmith et al. (2018); Granados‐Principal et al. (2011), Han et al. (2009), Luo et al. (2013), Sani et al. (2022), Sirianni et al. (2010), Sun et al. (2014), Terzuoli et al. (2016), Toteda et al. (2017), Zhao et al. (2014), Zubair et al. (2017)
Anti‐neurodegenerative diseases	APP/PS1 mice; N2a cells; astrocytic cell line C6; SH‐SY5Y cells; rat astrocytes, serum of MS patients; PC12 cells	Improvement of cognitive function; as neuroprotective agent against amyloid‐β‐induced toxicity; restoration of proper insulin signaling; protection of dopaminergic neurons against 6‐OHDA/induction of phase II detoxifying enzyme/inhibition of apoptosis; inhibition of gelatinases; decrease in oxidation of dopamine	Crespo et al. (2017), Goldstein et al. (2016), Peng et al. (2016), St‐Laurent‐Thibault et al. (2011), Yu et al. (2016)
Anti‐viral and anti‐microbial	Fungal strains: *A. nidulans*; *A. fumigatus*; *A. flavus; F. oxysporum*; *C. albicans*; *C. dubliniensis*. Bacterial strains: *P. aeruginosa*; *E. coli; Klebsiella* sp.; *P. fluorescens*; *S. aureus*; *B. subtilis*; *S. enterica*; *Yersinia*; *S. sonnei*. Viral strain: HIV‐1; SARS‐CoV‐2	High efficiency in fungal plasma membrane destruction and strong antifungal activity; HT involves effective penetration of cell membranes in both Gram‐negative and Gram‐positive bacteria	Bedoya et al. (2016), Bisignano et al. (1999), Crisante et al. (2016), Diallinas et al. (2018), Ergoren et al. (2020), Lee‐Huang et al. (2007), Medina et al. (2006), Zorić et al. (2016)
Anti‐adipogenic	3T3‐L1 preadipocyte cell	Upregulated genes involved in adipogenesis inhibition; downregulated genes promoting adipogenesis	Kurylowicz et al. (2015), Scoditti et al. (2015), Stefanon and Colitti (2016), Tutino et al. (2016)
Skin protection and wound healing	Melanoma cell; pulsed electromagnetic fields treated HUVECs; vascular endothelial cells	Decreased oxidative stress associated with UV radiation; promoting cell proliferation and inhibiting apoptosis; upregulates HO‐1 expression by stimulating the nuclear accumulation and stabilization of Nrf2	Almeida et al. (2014), Cheng et al. (2017), D'Angelo et al. (2005), Dunnill et al. (2017), Jindam et al. (2017), Krzyszczyk et al. (2018), Landén et al. (2016), Lawrence and Fong (2010), McGovern et al. (2011), Salucci et al. (2014), Schaible et al. (2013), Szabo et al. (2018), Wiegand et al. (2010), Zrelli et al. (2015), Zwane et al. (2012)

Currently gaining increasing attention from the food and health industries as a functional ingredient in yogurts, beverages, baked goods, and cooking oils, among other products, its market value is projected to surpass $2.5 billion by 2030, indicating significant growth potential. This review presents a comprehensive overview of recent advancements in the extraction, chemical synthesis, and biosynthesis of HT. Furthermore, this study delves into the pharmacological functions of HT, providing a robust theoretical foundation for its application.

## 2 THE SOURCES AND SYNTHETIC METHODS OF HYDROXYTYROSOL

### 2.1 Acquisition of natural resources for hydroxytyrosol

HT is a polyphenol that occurs naturally in various fruits and vegetables, with the highest abundance found in the olive plant as a byproduct of oleuropein degradation (Bartella et al., 2018). A considerable portion of the fruit's dry weight is composed of oleuropein, which is a phenolic compound found in significant quantities throughout the growth of olive plants. The complete release of HT requires hydrolysis measures, such as heat or acid treatments, subsequent to the breakdown of oleuropein either during the maturation process of fruits or after their cultivation (Ortega‐García & Peragón, 2010). In addition to olives, varying concentrations of HT can be found in grapes and wines depending on their type. Italian red wines have been reported to contain approximately 2.5–3.4 mg/kg^2^ of HT, while white wines contain approximately 0.2–2.3 mg/kg^2^ (Ragusa et al., 2017). Commercial extraction primarily focuses on olive sources because of the significant market demand for olives and olive oil. Byproducts such as olive oil mill wastewater (OMW) and two‐phase olive waste (alperujo), which are derived from the production of olive oil, are frequently employed for the extraction of HT compounds (Achmon & Fishman, 2015). OMW forms during the centrifugation process in olive oil milling, whereas alperujo refers to the semisolid waste fraction obtained from two‐phase olive oil processing (Fernández‐Bolaños et al., 2002). These waste sources retain up to 98%–99% of the phenolic content of olives after extraction processes, including HT (Robles‐Almazan et al., 2018). The ability to obtain HT naturally from olive oil and olive leaves promotes sustainability. Overall, natural sources such as olives, grapes, and their byproducts serve as potential resources for extracting HT. However, limitations to natural extraction have been noted; for example, HT is present at relatively low concentrations in olive oil and olive leaves. The concentration of HT in olive oil and olive leaves can vary, depending on factors such as the type of olive tree, location of the plantation, oil quality, and olive oil production process.

### 2.2 Physical extraction methods for hydroxytyrosol

HT can be obtained through the following physical extraction methods: ① Solvent extraction—The raw material containing HT (such as olive leaves, olive fruits, etc.) is mixed with an appropriate solvent (such as ethanol, ethyl acetate, etc.), stirred, and soaked to dissolve the HT in the solvent. Subsequently, HT is separated from the solution through filtration or centrifugation (Mulinacci et al., 2005). ② Ultrasonic extraction—The raw material containing HT is mixed with a solvent and subjected to ultrasonic waves to induce intense liquid vibrations and the formation of tiny bubbles, thereby accelerating the release and dissolution of HT. Ultrasonic extraction enhances both efficiency and speed (Xie et al., 2019). ③ Column chromatography separation—A solid‐phase material‐filled extraction column with hydrophilic properties or HT affinity is employed. The solution containing HT passes through the extraction column utilizing the adsorption and retention characteristics of the solid‐phase material for separating HT from other impurities. These methods can be optimized and combined according to specific requirements to obtain high‐purity HT at a high yield (Agalias et al., 2007). Physical extraction can achieve the efficient extraction of HT by selecting the appropriate solvent and extraction conditions to obtain high‐purity products. These methods are usually simpler than chemical synthesis or biosynthesis methods because the operation process is relatively easy to control and does not require complex reaction conditions or the involvement of enzymes. In addition, compared with biosynthetic methods that require expensive substrates, physical extraction methods can use inexpensive raw materials for extraction, reducing production costs.

However, physical extraction methods usually cannot achieve complete extraction, resulting in low yields. This outcome may be because the extraction conditions are not fully suitable for the extraction of HT or because other constraints exist that are difficult to overcome. Some physical extraction methods may require high‐temperature or high‐pressure conditions, which can lead to high energy consumption and increased production costs. The waste generated during the physical extraction process may contain hazardous substances, which need to be properly treated and disposed of to ensure environmental safety.

### 2.3 Chemical synthesis of hydroxytyrosol

The chemical synthesis of HT refers to the artificial production of HT through chemical reactions. In previous studies, HT was synthesized by reducing 3,4‐dihydroxyphenylacetic acid (DOPAC) using lithium aluminum hydride (LiAlH_4_) as a catalyst, resulting in a productivity of 1.93 g/L/h (Britton et al., 2019). However, this method was not further developed due to the exorbitant cost of the substrate and the highly reactive nature of LiAlH_4_. In a more recent study, a modified chemical synthesis method involving a reflux reaction with tetrahydrofuran (THF) as the solvent was employed, resulting in the conversion of tyrosol or homovanillyl alcohol to carboxymethylated HT with an overall yield of 72% (Bernini et al., 2008). The advantage of this method lies in its use of commercially available substrates and environmentally friendly solvents. Several other chemical synthetic methods for producing HT have been developed: ① The Baeyer–Villager oxidation (BVO) method involves treating tyrosol with ptoluenesulfonic acid in ethyl acetate (EA), leading to the formation of tyrosol acetate. Subsequently, orthoformyl‐tyrosol acetate is generated by reacting tyrosol acetate with paraformaldehyde. Finally, orthoformyl‐tyrosol acetate reacts with meta‐chloroperoxybenzoic acid (MCPBA) to produce HT at an overall yield of 60%, resulting in a productivity of 0.33 g/L/h (Piersanti et al., 2011). ② In the eugenol method: eugenol, derived from clove oil, is combined with ethanol and sodium borohydride to produce homovanillyl alcohol. Homovanillyl alcohol then undergoes a reaction with sodium iodate to generate HT at an overall yield of 76.5%, for productivity of 0.69 g/L/h (Deffieux et al., 2014). ③ In the catechol method, catechol, a widely available compound, is hydroxylated using 2,2‐dimethoxy acetaldehyde to form 4‐(hydroxyalkylacetal)‐catechols which are subsequently reduced to HT, yielding an overall efficiency rate of 31.3% and a productivity of 0.29 g/L/h (Ziosi et al., 2018). ④ The DOPAC method involves the utilization of an undisclosed precursor molecule by Seprox, a Spanish company, to synthesize DOPAC as the initial compound. Subsequently, DOPAC is subjected to esterification and reduction processes to yield crude HT, which undergoes further purification steps to obtain the final HT product (Britton et al., 2019).

In general, chemical synthesis offers an alternative approach for obtaining HT from natural sources, enabling the controlled and efficient production of this compound. However, some chemical synthesis methods require expensive substrates, such as tyrosol, which can increase the cost of HT production. Certain chemical synthesis methods may require harsh reaction conditions, such as high temperatures or strong acids, which can lead to increased energy consumption and potential safety concerns. Overall, while chemical synthesis methods offer advantages such as high purity and scalability, they may also have drawbacks such as expensive substrates and the use of heavy metal catalysts. Improving the yield and cost‐effectiveness still poses challenges that need to be addressed.

### 2.4 Biocatalysis of hydroxytyrosol

The biotechnological production of HT involves the use of biological methods, such as enzymes or microorganisms, to synthesize HT. The aim is to develop a cost‐effective process with high efficiency and productivity utilizing an economical starting material. Two primary strategies for substrate selection in biotechnological production have been identified: employing a structurally analogous substrate to HT or utilizing a simple carbon source such as glucose. One approach entails the use of structurally similar precursors, such as 2‐phenylethanol or 3‐nitrophenethyl alcohol, which can be converted into HT through fewer enzymatic steps (Bernath‐Levin et al., 2014; Brouk & Fishman, 2009). Several pathways exist for the biocatalytic production of HT. One method involves whole‐cell biosynthesis by converting tyrosine into HT using enzymes. Another approach focuses on modifying the shikimate pathway to enhance carbon flux toward HT synthesis. In terms of yield, the whole‐cell biosynthetic method achieves relatively low titers and yields of HT from glucose alone, with a productivity rate of 0.01 g/L/h when no additional substrate was supplemented. However, supplementation with exogenous tyrosine or L‐DOPA significantly increases both productivity and yield. The highest yield (74%) is attained when L‐DOPA is added as a supplement to the culture (Satoh et al., 2012). Modification of the shikimate pathway results in a final titer of 0.65 g/L when media containing glucose are used and the titer reaches 1.24 g/L upon supplementation with tyrosine (Li et al., 2018). When glucose and glycerol are the only substrates used, the overall productivity rate for HT is 0.01 g/L/h, but it increases to 0.03 g/L/h when tyrosine is added (Satoh et al., 2012). In a study conducted by Chung et al. (2017), plant genes were inserted into *E. coli* to produce HT from tyrosine. Two distinct pathways have been employed for the conversion of tyrosine to HT. One pathway utilizes an enzyme derived from plants, known as aromatic acetaldehyde synthase (AAS), to directly convert tyrosine to 4‐hydroxyphenylacetaldehyde (4HPAA). The other pathway involved the decarboxylation of tyrosine to tyramine, followed by oxidation to 4HPAA. Subsequently, 4HPAA is converted to tyrosol and further transformed into HT. The results indicate that the highest production of HT reaches 0.2 g/L during a growth period of 30 h, with a yield lower than 0.01 g/L/h. This reduced yield could be attributed to the utilization of unoptimized plant‐derived genes (Li et al., 2018).

Substrates structurally analogous to HT, such as 2‐phenylethanol, 3‐nitrophenylethanol, DOPAC, and oleuropein, have been employed. By employing enzymes or enzyme variants, these substrates can be converted into HT in fewer enzymatic steps. This strategy has the potential for enhancing HT production compared to the use of glucose or tyrosine as starting materials. For instance, Brouk and Fishman utilized a modified enzyme called toluene monooxygenase (T4MO) for the conversion of 2‐phenyloxanol to HT (Brouk & Fishman, 2009). The yield achieved in small‐scale reactions was 10%, which was subsequently increased to 48% in large‐scale reactions (Brouk & Fishman, 2012). Similarly, Bernath‐Levin et al. employed a modified form of nitrobenzene dioxygenase to catalyze the conversion of 3‐nitrophenyl alcohol to HT, resulting in a production rate of 0.12 g/L/h (Bernath‐Levin et al., 2014). Other studies have investigated the utilization of alternative precursors, such as DOPAC and oleuropein, for the production of HT (Santos et al., 2012). These studies have reported varying degrees of success in terms of HT yield and productivity.

HT is also generated through biocatalysis using purified enzymes or cell‐free extracts. This method involves the utilization of biocatalysts, such as tyrosinase, to convert tyrosol into HT (Espín et al., 2001). Tyrosinases are enzymes that have the ability to convert monophenols into diphenols through hydroxylation and to oxidize O‐diphenols into O‐quinones. During HT production, tyrosol undergoes an initial transformation into HT through the monophenolase activity of tyrosinase. This step is then followed by the reduction of orthoquinone to HT using ascorbic acid as a reducing agent (Espín et al., 2001). This process has generated high productivity levels, achieving a 90% conversion yield to HT within 4 h using a 16 mM tyrosol matrix (Espín et al., 2001). Other studies have investigated the use of different tyrosinases from various sources, including fungi and bacteria, for converting tyrosol into HT (Halaouli et al., 2005). The biocatalytic synthesis of HT offers several advantages. It allows for the production of HT using renewable resources, such as microorganisms or plant extracts, which can be more sustainable than to chemical synthesis methods. Biocatalysis also offers the potential for higher selectivity and specificity, resulting in a purer product. Additionally, biocatalytic processes can often be performed under mild reaction conditions, reducing the need for harsh chemicals or high temperatures. In a recent study by O'Connor et al., a tyrosinase derived from *Ralstonia solanacearum* was employed for the conversion of tyrosol to HT. The enzyme was heterologously expressed in an *E. coli* host and utilized in both purified and crude lysate forms. The purified enzyme exhibited a conversion efficiency of 100% for 75 mM tyrosol within a 90 min timeframe, resulting in a productivity rate of 7.7 g/L/h, thus establishing it as the most efficient biocatalyst for HT production to date (Kevin et al., 2017). This method can lead to lower energy consumption and a reduced environmental impact. Furthermore, biocatalysis can be more cost‐effective in the long run, as it can utilize inexpensive carbon sources and enzymes can be reused multiple times. However, utilizing tyrosol as a substrate can be costly and limits the potential scalability of this biocatalytic approach. Future research in this field could focus on developing artificial pathways for the efficient production of HT from more readily available and inexpensive carbon sources, such as glucose and tyrosine. Additionally, efforts aimed at enhancing enzyme efficiency while minimizing product toxicity toward host organisms can further enhance the biotechnological yield of HT.

## 3 BIOLOGICAL ACTIVITY OF HYDROXYTYROSOL

### 3.1 Evaluation of the pharmacokinetics, pharmacodynamics, and safety profile

The dose‐dependent absorption of HT from olive oil following ingestion has been indicated to have noteworthy biological impacts (Serreli & Deiana, 2018). HT undergoes hydrolysis in the small intestine, resulting in the presence of oleuropein as a free form in the serum. Additionally, the intestinal flora decomposes oleuropein into HT (Corona et al., 2006; Hu et al., 2014). HPLC analysis revealed that the enzymatic conversion of HT can involve oxidative and/or methylation reactions, leading to the formation of its O‐methylated derivative, glucuronate conjugate, and glutathione conjugate (de Bock et al., 2013). Furthermore, studies conducted on mouse and rat models have shown that even at doses up to 2000 mg/kg, HT possesses a favorable safety profile with no observed side effects (Christian et al., 2004; Soni et al., 2006).

Due to its remarkable biological properties mentioned above, HT is being extensively researched as one of the most prominent natural phenols. Its exceptional safety profile renders it an optimal dietary supplement for the nutrition and healthcare sectors.

### 3.2 Antioxidant effect

The hydroxyl group of HT at the ortho position reveals that the olive extract exhibits the highest antioxidant activity among the various extracts due to its exceptional electron donor ability and the formation of a stable hydrogen bond with the phenoxy group (de la Torre‐Carbot et al., 2005; Fitó et al., 2005). In vitro studies have shown that even at very low concentrations, HT exhibits a protective effect on the oxidation of low‐density lipoprotein (LDL) (Rietjens et al., 2007). Furthermore, HT not only scavenges oxidative chemicals but also stimulates antioxidant enzyme activity and synthesis, thereby enhancing overall antioxidant effects. Notably, antioxidants in the body can function as either reactive oxidants or electrophilic antioxidants. The beneficial effect of prooxidation relies on achieving an adequate concentration in vivo to activate signaling pathways recognizing free radicals (Forman et al., 2014). By exerting its antioxidant activity, HT effectively prevents oxidative damage to cellular macromolecules such as DNA and reduces oxidative stress. Both in vitro and in vivo studies have shown that it efficiently eliminates reactive oxygen species (ROS) and decreases their levels. Additionally, HT enhances the plasma total antioxidant capacity by increasing the body's overall antioxidant capability (Fuccelli et al., 2018). Notably, HT induces Nrf2 synthesis and translocation into the nucleus, facilitating the transcription of multiple genes that encode proteins responsible for the antioxidant response, such as proteins involved in DNA repair or enzymes associated with type II detoxification (Zrelli, Matsuoka, Kitazaki, Araki, et al., 2011).

### 3.3 Anti‐inflammatory effects

Inflammation serves as a defensive response to cellular damage, stimuli, or pathogens and involves the participation of both immune cells and molecular mediators. However, currently available anti‐inflammatory medications (including statins, glucocorticoids, nonsteroidal anti‐inflammatory drugs, and disease‐modifying antirheumatic drugs) not only pose potential risks of toxicity but also fail to address the underlying inflammatory conditions (Kapoor et al., 2014).

HT exerts its anti‐inflammatory effects through diverse mechanisms. It has been shown to modulate inflammation and autophagy or other crucial processes involved in the regulation of inflammation. In terms of inflammation modulation, HT has been found to decrease the secretion of proinflammatory cytokines such as interleukin‐1 beta (IL‐1β), interleukin‐6 (IL‐6), and tumor necrosis factor‐alpha (TNF‐α) in various in vivo models (Ramírez‐Expósito & Martínez‐Martos, 2018). As a result, it inhibits the pathways of inducible nitric oxide synthase/nitric oxide and cyclooxygenase/prostaglandin E2 (Borrie & Kim, 2017; Martínez et al., 2019). The results of one study indicated that when HT is orally administered to mice with pristane‐induced systemic lupus erythematosus, a notable decrease is observed in the secretion of IL‐1β and IL‐6 by splenocytes and macrophages stimulated with LPS ex vivo (Aparicio‐Soto et al., 2017). Moreover, HT suppresses the stimulation of nuclear factor‐kappa B (NF‐κB), a crucial transcription factor responsible for controlling inflammation. NF‐κB governs the expression of various genes implicated in inflammation, including those encoding proinflammatory cytokines and adhesion molecules (García‐García et al., 2021; Tavenier et al., 2020). By preventing the degradation of inhibitor of nuclear factor‐kappa B (IκB), NF‐κB p65 nuclear translocation is inhibited, and MAPK phosphorylation is inhibited (Gallardo‐Fernández et al., 2019). HT effectively inhibits NF‐κB activation to reduce the overall inflammatory response. In an LPS‐induced acute liver injury model, HT decreased the number of inflammatory M1/CD11c^+^ macrophages while increasing the number of anti‐inflammatory M2/CD206^+^ macrophages within liver tissue. Additionally, it reduced liver TNF‐α, IL‐lβ, and IL‐6 mRNA levels while elevating serum IL‐10 and IL‐4 protein levels (Yu et al., 2020). These findings suggest that by modulating macrophage‐mediated inflammation, HT mitigates hepatic inflammation and injury (Yu et al., 2020). In atherosclerosis models, the administration of HT resulted the extent of aortic atherosclerotic lesions and the serum levels of CRP, TNF‐α, IL‐1β, and IL‐6 while increasing IL‐10 levels. Moreover, HT attenuated the activation of inflammatory signaling molecules such as phosphorylated p38 MAPK and activated NF‐κB in the liver, indicating that HT has an antiatherosclerotic effect through the inhibition of inflammatory signaling molecules (Zhang, Qin, et al., 2020). Additionally, HT inhibited neuroinflammation in vivo. Pretreatment with HT followed by LPS administration significantly decreased LPS‐induced changes in the mRNA levels of IL‐6, IL‐1β, and TNF‐α in the brain (Zhang, Zhang, et al., 2020). Furthermore, HT alleviated neuropathic pain in rats by reducing the production of inflammatory cytokines within the spinal dorsal horn and inhibiting ERK signaling pathway activation (Yu et al., 2022). Notably, HT also diminishes the infiltration of immune cells, including macrophages and neutrophils, into inflamed tissues. This effect is mediated by the downregulation of adhesion molecules involved in immune cell recruitment to inflamed tissues, such as intercellular adhesion molecule 1 (ICAM‐1) and vascular cell adhesion molecule 1 (VCAM‐1) (Catalán et al., 2015). These results suggest that at nutritionally appropriate concentrations, HT exerts suppressive effects on matrix metalloproteinase (MMP)‐9 and cyclooxygenase‐2 (COX‐2) enzyme activity/expression in activated human monocytes via the inhibition of PKCα/PKCβ1, resulting in the manifestation of anti‐inflammatory effects (Scoditti et al., 2014). In summary, HT exhibits anti‐inflammatory activity through the suppression of proinflammatory cytokine production, NF‐κB activation, and a reduction in immune cell infiltration into inflamed tissues. These mechanisms contribute to the overall anti‐inflammatory effects exerted of HT.

### 3.4 Treatment of neurodegenerative diseases

Monoamine oxidase (MAO) inhibition is believed to decelerate the progression of Parkinson's disease (PD) by reducing the production of the autotoxic dopamine metabolite 3,4‐dihydroxyphenylacetaldehyde. However, it generates toxic dopamine oxidation products such as 5‐S‐cysteine‐dopamine. HT has been reported to exert neuroprotective effects by diminishing the levels of monoamine oxidase inhibitor products (Goldstein et al., 2016). The potential role of HT in neurodegenerative disorders lies in its ability to activate the Nrf2/ARE pathway, which serves as a pivotal neuroprotective pathway. HT induces the nuclear translocation of Nrf2 and increases the expression and activity of antioxidant enzymes (Zou et al., 2012). It has been reported to augment the activity of GSH‐related enzymes and induce heme oxygenase‐1 (HO‐1) expression in various cell types (Zrelli, Matsuoka, Kitazaki, Araki, et al., 2011). Furthermore, HT activates the PI3K/Akt and ERK1/2 pathways, which are implicated in inducing antioxidant enzymes (Martín et al., 2010). Additionally, HT has been found to increase nuclear factor levels of Nrf2 while decreasing nuclear levels of Bach1, a transcription factor that represses antioxidant‐responsive element (ARE) sequences (Sgarbossa et al., 2012). In vivo studies conducted using a mouse model exhibiting early learning and memory decline revealed that HT can activate Nrf2 and increase antioxidant enzyme expression. These findings suggest that HT may confer beneficial effects on neurodegenerative diseases through Nrf2 activation (Arunsundar et al., 2015). Moreover, HT attenuates microglial activation induced by LPS and α‐synuclein, indicating its potential for anti‐inflammatory and anti‐neurodegenerative applications (Gallardo‐Fernández et al., 2019).

Overall, HT has shown preventive effects on the development of neurodegenerative disorders such as Alzheimer's disease (AD) and PD through its anti‐inflammatory and antioxidant properties. HT exerts neuroprotective effects by attenuating lactate dehydrogenase efflux, suppressing NF‐κB activation, modulating antioxidant enzyme levels, ameliorating brain inflammation and mitochondrial oxidative stress, and enhancing the insulin signaling pathway. These mechanisms collectively contribute to the protective effects of HT on AD and PD (Gorzynik‐Debicka et al., 2018; Silva et al., 2023). However, further investigations are still underway to elucidate the specific mechanisms underlying the HT‐mediated reduction in neurodegeneration in AD and PD patients.

### 3.5 Effects on cancer cells

The inhibitory effect of HT on cancer cell proliferation has been documented in studies. HT decreases the quantity of Michigan Cancer Foundation‐7 (MCF‐7) cells in a breast cancer cell model, and HT reduces the number of MCF‐7 cells by suppressing cell proliferation and inducing apoptosis (Chimento et al., 2014; Han et al., 2009). The antiproliferative effect of HT on MCF‐7 cells is not mediated through ERα‐regulated gene mechanisms but rather through the inhibition of E2‐induced activation of extracellular regulated kinase 1/2 (ERK1/2), a member of the mitogen‐activated protein kinase family (Sirianni et al., 2010). This effect was also observed on ER‐negative SK‐BR‐3 breast cancer cells, where HT induced apoptosis through the antagonism of G protein‐coupled estrogen receptor (GPER) (Chimento et al., 2014). Additionally, HT inhibited rat mammary tumor growth and proliferation similarly to doxorubicin while promoting high expression of Sfrp4, a gene associated with cell proliferation and the Wnt signaling pathway (Granados‐Principal et al., 2011). HT has also been investigated in various types of cancer cells including colon cancer cells, where it triggers apoptosis by generating H_2_O_2_ and ROS. This mechanism may be linked to dysregulated expression of catalase, an enzyme responsible for converting H_2_O_2_ into water and oxygen within cancer cells (Sun et al., 2014). HT induces apoptosis in colorectal cancer cells by triggering cell cycle arrest (Sani et al., 2022). In the pancreatic cancer cell line MIA PaCa‐2, HT induces cell cycle arrest in G2 phase and affects cell cycle progression. Furthermore, HT promotes apoptotic death accompanied by an increase in the Bax/Bcl‐2 ratio followed by caspase 3/7‐dependent programmed cell death suggesting its potential as a therapeutic agent against pancreatic cancer (Goldsmith et al., 2018). HT induces apoptosis and mitochondrial dysfunction in prostate cancer PC‐3 cells via superoxide production (Luo et al., 2013). Moreover, it facilitates apoptotic cellular death through intrinsic pathways, thereby reducing viability among thyroid cancer cells (Toteda et al., 2017). Previous studies have documented the ability of HT to induce apoptosis, a process of programmed cell death, specifically in leukemia cells, while having no impact on normal cells (Fabiani et al., 2002). The findings revealed that HT exerts its potential anticancer effect through the inhibition of the Akt, NF‐κB, STAT3, and EGFR signaling pathways, thereby suppressing cancer cell proliferation across various cancer cell lines (Gonçalves et al., 2024; Terzuoli et al., 2016; Zhao et al., 2014; Zubair et al., 2017).

### 3.6 Antiviral and antimicrobial effects

The in vitro anti‐HIV activity of HT has been reported in multiple studies, as it effectively inhibits viral integrase activity and the fusion of the viral envelope with host cells (Lee‐Huang et al., 2007). HT exhibits inhibitory effects on HIV‐1 infection in target cells, regardless of whether it is a recombinant or wild‐type virus (Bedoya et al., 2016). Although HT is not considered a potent oral antiretroviral therapy (ART) due to its low intrinsic activity, it can inhibit the replication of various HIV‐1 strains and resistant isolates in vivo without any deleterious effects, either additively or synergistically with other antivirals (Lee‐Huang et al., 2007). Moreover, HT induces morphological changes that reduce influenza virus infectivity and enhance anti‐inflammatory effects by reducing the levels of the proinflammatory cytokines IL‐6 and TNF‐α. These properties may hold promise for combating severe acute respiratory syndrome coronavirus 2 (SARS‐CoV‐2) (Ergoren et al., 2020).

Since the 1970s, substantial evidence supporting the antibacterial and antimicrobial properties of olive extracts has emerged. HT exhibits notable antimicrobial activity against *Candida albicans* and *Candida dubliniensis* (Zorić et al., 2016). Recent investigations have revealed its potent efficacy against *Clostridium perfringens, E. coli, Staphylococcus aureus, Salmonella enterica, Yersinia*, and *Shigella sonnei* (Crisante et al., 2016; Medina et al., 2006). The mechanism of action underlying HT involves the effective penetration of cell membranes in both Gram‐negative and Gram‐positive bacteria, potentially leading to the disruption of cell peptidoglycans or membrane damage (Bisignano et al., 1999). Consequently, HT holds promise as a potential microbicide.

### 3.7 Antiadipogenic effect

The role of HT in the regulation of obesity is crucial, particularly in its ability to prevent the suppression of adiponectin expression induced by TNF‐α. Adiponectin, a distinct protein released by adipocytes, has been shown to combat diabetes, inflammation, and atherosclerosis (Scoditti et al., 2015). HT suppresses the expression of the cannabinoid CB1 receptor gene in the 3T3‐L1 preadipocyte cell line, suggesting its potential anti‐obesity effects through modulation of the endocannabinoid pathway. Moreover, HT inhibits cell proliferation and adipogenesis in 3T3‐L1 cells (Kurylowicz et al., 2015; Tutino et al., 2016). Treatment with HT significantly upregulates genes involved in inhibiting adipogenesis (*GATA2, GATA3, WNT3A, SFRP5, HES1, and SIRT1*) in primary human visceral adipocytes. Conversely, it downregulates genes promoting adipogenesis (*LEP, FGF1, CCND1, and SREBF1*) (Stefanon & Colitti, 2016). Additionally, during the primary human visceral preadipocyte differentiation process, HT promotes lipolysis and apoptotic activities but has no effect on mature adipocytes (Kurylowicz et al., 2015; Stefanon & Colitti, 2016). Therefore, HT has a protective effect on fat accumulation and obesity while preventing diseases associated with these factors.

### 3.8 Skin protection and wound healing

The protective effects of HT on the skin, including a reduction in skin tumor incidence and protection against UV radiation‐induced damage, have been documented. HT has been shown to decrease oxidative stress associated with UV radiation and exhibit cytotoxicity toward melanoma cells exposed to UVA radiation (D'Angelo et al., 2005). Furthermore, it exhibits a significant shielding capacity against the negative effects caused by UVB (Salucci et al., 2014). Moreover, a novel derivative of HT has exhibited high efficacy in combating oxidative stress induced by UV radiation and plays a role in modulating apoptosis (Zwane et al., 2012).

HT has been reported to possess various specific mechanisms of action in the process of wound healing. Oxidative stress occurs when an imbalance between the generation of ROS and the body's capacity to neutralize them using antioxidants exists. HT plays a crucial role in reducing oxidative stress in chronic wounds (Dunnill et al., 2017). HT can effectively regulate ROS production and activate Nrf2 and HO‐1, both of which are involved in antioxidant defense mechanisms (Jindam et al., 2017; Szabo et al., 2018). Moreover, HT possesses potent anti‐inflammatory properties that are essential for promoting wound healing. While inflammation is a normal response to injury, prolonged inflammation can impede the healing process (Landén et al., 2016). HT aids in reducing the production of proinflammatory cytokines and facilitates the action of anti‐inflammatory cytokines (Wiegand et al., 2010). Additionally, it inhibits the activity of NF‐κB, a transcription factor responsible for regulating inflammatory responses (Lawrence & Fong, 2010). Furthermore, HT demonstrates remarkable antimicrobial activity that is pivotal for preventing and treating infections associated with wounds (Krzyszczyk et al., 2018). Given that chronic wounds are susceptible to bacterial colonization, HT effectively inhibits the growth of bacteria such as *Staphylococcus* species, *Streptococcus* species, *Pseudomonas aeruginosa*, and *E. coli* (Almeida et al., 2014; McGovern et al., 2011; Schaible et al., 2013). This antimicrobial effect contributes significantly to creating an optimal environment conducive to wound healing. Overall, these specific mechanisms, encompassing antioxidant, anti‐inflammatory, and antimicrobial activities, establish HT as a promising bioactive compound suitable for applications in wound care.

## 4 DISCUSSION AND CONCLUSION

Extracting large amounts of HT from natural resources, such as the byproducts generated during olive oil production, is feasible. However, due to the complex extraction process and low yield, the cost of this process becomes prohibitive. Furthermore, HT obtained from natural sources exhibits low purity levels and is subject to seasonal and batch variations, posing challenges in achieving consistent industrial benefits (Britton et al., 2019). HT can also be obtained through other methods, including chemical, physical, and biotechnological approaches. Chemical extraction techniques for HT involve the use of solvents and chromatographic methodologies to separate HT from other phenolic compounds present in olive extracts (Capasso et al., 1992; Mulinacci et al., 2005). Chromatographic separation utilizing reverse‐phase chromatographic columns has effectively isolated HT from other compounds within the mixture (Xynos et al., 2015). Physical extraction methods entail the use of supercritical CO_2_ or resins to isolate HT from olive sources (Bartella et al., 2021). Chemical synthesis provides an alternative approach for acquiring HT from natural sources, facilitating the precise and efficient production of this compound. However, drawbacks have been noted such as the use of costly substrates and heavy metal catalysts, which exert pressure on the environment (Ziosi et al., 2018). The whole‐cell biocatalytic production of HT is a promising approach for generating this potent antioxidant (Satoh et al., 2012). Biocatalytic synthesis is a flexible way to improve optimization. The production of HT through artificial pathways using inexpensive substrates is very attractive, but it still faces the problems of low yield and low concentration (Chen et al., 2019; Trantas et al., 2019). HT has been used as a drug and food additive; however, due to its low extraction rate and high price, currently, the supply in the market is insufficient (Achmon & Fishman, 2015; Robles‐Almazan et al., 2018). A high industrial production scale helps to promote the application of HT. Therefore, the production efficiency and the purity are problems that need to be solved in the future. Through metabolic pathway modification, the use of structurally analogous precursors, and protein engineering techniques, efforts are being made to enhance enzyme performance and mitigate the host's biological toxicity associated with product formation. This approach may represent a prevailing trend in advancing future HT biotechnology production.

The inclusion of extra virgin olive oil, a rich source of the phenolic compound HT, is one of the key components in the renowned Mediterranean diet and is known for its numerous health benefits (Rodríguez‐Morató et al., 2016). HT has been found to possess multiple pharmacological activities, including antioxidant, anti‐inflammatory, and pro‐apoptotic effects (Fabiani et al., 2012; Fernández‐Prior et al., 2021). It has been shown to confer protection against the aging process through the activation of AMPK and promotion of autophagy (de Pablos et al., 2019). AMPK serves as a cellular energy sensor that regulates diverse metabolic processes, including glucose uptake and fatty acid oxidation (Garcia & Shaw, 2017). The activation of AMPK by HT can enhance cellular energy metabolism and mitigate oxidative stress, which are pivotal factors in the aging process (Zrelli, Matsuoka, Kitazaki, Zarrouk, & Miyazaki, 2011). Furthermore, investigations have explored the effects of HT on various age‐related diseases. HT also exerts neuroprotective effects by reducing oxidative stress, inflammation, and β‐amyloid‐induced toxicity. Moreover, it can enhance cognitive function and restore proper insulin signaling, which are crucial for preventing the development of diseases such as AD and PD (Hadrich et al., 2022; Romero‐Márquez et al., 2023). Overall, HT has promising anti‐aging effects and protective effects on various age‐related diseases due to its antioxidant properties in addition to its anti‐inflammatory and pro‐apoptotic activities. In the context of osteoporosis, HT has been shown to confer protective effects on bone health through its ability to promote osteoblast differentiation and inhibit of osteoclast activity (Hagiwara et al., 2011). Furthermore, it can effectively attenuate oxidative stress and inflammation within bone cells. Further investigations are warranted for a comprehensive understanding of its mechanisms of action, as well as for exploring its potential application as an anti‐aging therapy. In cancer, HT has been reported to exert antitumor effects by impeding cell proliferation and inducing apoptosis in cancer cells (Imran et al., 2018). Additionally, it possesses the ability to modulate signaling pathways implicated in cancer development and progression (Imran et al., 2018). In metabolic syndrome, HT has the potential to ameliorate insulin sensitivity and mitigate inflammation, both of which are pivotal factors contributing to the onset of metabolic disorders such as obesity and type 2 diabetes (Peyrol et al., 2017). In immune‐mediated diseases, HT has displayed immunomodulatory properties along with a capacity to suppress inflammatory responses by inhibiting proinflammatory cytokine production while promoting anti‐inflammatory cytokine generation (Santangelo et al., 2018). HT has also been considered a potential microbicide (Bedoya et al., 2016). Due to its antioxidant, anti‐inflammatory, and antimicrobial properties, HT plays a role in skin protection and wound healing (Abate et al., 2021; Batarfi et al., 2023). In addition, the European Food Safety Authority has officially verified the safety of HT (Turck et al., 2017). In conclusion, the legal status of HT as a novel food ingredient approved for addition to a wide range of foods in accordance with the implementing decision of the European Commission opens the possibility of expanding its use in other foods in the future (Gallardo‐Fernández et al., 2022).

In this review, we present a comprehensive overview of the methodologies employed for HT extraction. We discuss the differences between the various production methods and the development trends of future production strategies. HT has been extensively investigated due to its remarkable biological activities and beneficial effects on human health. This review substantiates previous indications and reveals novel facets and the therapeutic potential of HT. HT is expected to be a promising food additive and a potential therapeutic agent.

## AUTHOR CONTRIBUTIONS

**Enhui Wang:** Investigation (lead); writing – original draft (equal); writing – review and editing (equal). **Yanfei Jiang:** Conceptualization (equal); funding acquisition (lead); supervision (lead); writing – review and editing (supporting). **Chunyue Zhao:** Conceptualization (equal); writing – original draft (equal); writing – review and editing (lead).

## FUNDING INFORMATION

This work is supported by Beijing Nova Program (20220484186) to Yanfei Jiang, Beijing Municipal Science & Technology Commission.

## CONFLICT OF INTEREST STATEMENT

The authors have no conflicts of interest to declare.

## ETHICS STATEMENT

It is not applicable because this study is based exclusively on published literature.

## Data Availability

Data sharing is not applicable to this article as no new data were created or analyzed in this study.
